# Supplementary material for: Effects of harvest stages and lactic acid bacteria additives on the nutritional quality of silage derived from triticale, rye, and oat on the Qinghai-Tibet Plateau
Source: PeerJ. 2023 Aug 3;11:e15772. doi: 10.7717/peerj.15772 (PMC10404394; doi:10.7717/peerj.15772)
Supplement: Supplemental Information 2 [file peerj-11-15772-s002.docx]

Sila-Max contains purified amylase and cellulase as well as *Pediococcus acidilactici, Enterococcus faecium*, and *Propionibacterium acidipropionici*. Sila-Mix contains calcium silicate, *Lactobacillus plantarum, P. acidilactici, E. faecium, P. acidipropionici,* and *Aspergillus niger*.
